# The Current Status of Frailty and Influencing Factors in Elderly Patients With Hip Fractures: A Meta-Analysis

**DOI:** 10.1155/bmri/7756605

**Published:** 2025-04-13

**Authors:** Kangquan Shou, Zhuoqing Wu, Zuyang Xi, Longtao Zhao, Chaxiang Li, Tongzhu Bao, Jianghua Lv, Youdan Shi

**Affiliations:** ^1^Department of Orthopedic, Yichang Central People's Hospital & The First College of Clinical Medical Science, China Three Gorges University, Yichang, China; ^2^Nursing Faculty, Yangtze University Health Science Center, Jingzhou, China; ^3^Department of Nursing, Yichang Central People's Hospital & The First College of Clinical Medical Science, China Three Gorges University, Yichang, China; ^4^Department of Orthopedic, Zhijiang People's Hospital, Yichang, China

**Keywords:** elderly, frailty, hip fractures

## Abstract

**Background:** Hip fractures have emerged as a significant health risk, posing a substantial threat to the well-being and longevity of the elderly population. The occurrence of postoperative complications and frailty profoundly impacts the quality of life in these individuals. This meta-analysis is aimed at elucidating the present scenario and clarifying the current status and influencing factors of frailty in elderly hip fracture patients. The findings will serve as a solid evidence for formulating effective and scientific strategies to prevent frailty in this vulnerable patient group.

**Methods:** The databases, including PubMed, Cochrane Library, and Embase, were utilized from February 2003 to February 2023, using the keywords frailty, elderly, and hip fractures to identify systematic reviews or meta-analyses. The primary randomized clinical trials included in systematic reviews or meta-analyses were identified. Two independent authors assessed the quality of all eligible studies. Statistical analyses were evaluated using Review Manager (RevMan) Version 5.3.

**Results:** Of the 15 studies out of 352 identified studies, 12 cross-sectional research studies, 2 case–control studies, and 1 cohort study were confirmed. There were 3475 hip fracture patients, 1209 of them showed frailty, with a 41% incidence of frailty, and 18 influence factors were determined. A marked between-study heterogeneity (*I*^2^ = 95%, *p* < 0.00001) was examined. Factors influencing frailty in elderly hip fracture patients were identified including age (odds ratio (OR) = 2.73, 95% confidence interval (CI): 2.12~3.53), comorbidity (OR = 4.20, 95% CI: 2.31~7.62), duration of bed rest (OR = 2.22, 95% CI: 1.54~3.18), nutritional status (OR = 1.62, 95% CI: 1.21~2.17), and self-perceived health status (OR = 3.53, 95% CI: 2.09~5.94). There was no publication bias, and the pooled results were stable basing on sensitivity analysis.

**Conclusion:** Frailty in elderly hip fracture patients is associated with a variety of factors consisting of age, comorbidity conditions, extended periods of bed rest, nutritional status, high comorbid conditions, poor self-perceived health status, advanced age, and poor nutritional status.

## 1. Introduction

With the aging of the population, hip fractures have evolved into a crucial health risk for the elderly, increasingly serving as one of the predominant reasons for their hospitalization [1, 2]. It has a high annual incidence of approximately 75,000 (mean age, 83~84) steadily increasing and expected to reach 6.3 million individuals by the year 2050 [3]. Frailty is a common syndrome associated with poor health outcomes in older replacement patients [4]. Recent studies focus on investigating the postoperative occurrence of frailty. It showed that the incidence of frailty in elderly hip fracture patients was high, ranging from 40.77% to 86.6% [5–7]. In addition, the occurrence of frailty is likely to promote the development of postoperative complications in patients, affecting their prognosis and regression [8, 9]. Moreover, Kua et al. [10] used different frailty measurement tools in 82 surgical patients, and the incidence of frailty was found to vary basing on the measurement tool. The Modified Fried Criteria (MFC) scale indicated a high frailty incidence of 86.6%, while the Reported Edmonton Frail Scale (REFS) suggested a considerably lower rate at 34.1%. Some studies proved a difference in the occurrence rate of frailty among geriatric patients suffering from femoral neck fractures, with a discrepancy between community-dwelling and hospitalized individuals [11–13]. In a study conducted by Huang and Lan [14], it was found that the prevalence of frailty in elderly patients with hip fractures was 40.91% at the time of admission, increasing to 46.75% upon discharge, suggesting that the prevalence of frailty in these patients varies across different stages, potentially influencing their functional recovery and quality of life. Additionally, a study found that individuals with possible sarcopenia had a significantly higher risk of hip fractures than those without possible sarcopenia (hazard ratio: 2.00, 95% confidence interval (CI): 1.46–2.75; *p* < 0.001) [15], which shows the prevalence of sarcopenia is very high, and sarcopenia is a significant predictor of adverse outcomes in patients with hip fractures [16]. In order to resolve the frailty in elderly patients, some studies demonstrated that the adoption of nutritional intervention and exercise intervention as well as comprehensive intervention is able to improve the frailty status of elderly patients effectively, thus enhancing their quality of life [17]. Although there are a number of studies investigating on the frailty related to the elderly patients, there is no integrated analysis of the current situation of its occurrence, thus failing to reflect the coexisting influencing factors of this group. Furthermore, there is no individualized intervention program yet to resolve the current situation of the patients' frailty. Therefore, we conducted a meta-analysis to scrutinize the prevalence and determinants of frailty among elderly patients with hip fractures. The aim of the present study is to elucidate the intrinsic associations, thereby providing theoretical support for clinical healthcare professionals to identify high-risk groups at early stage and designate corresponding intervention measures.

## 2. Data and Methods

### 2.1. Search Strategy

Two independent researchers systematically explored the following both English and Chinese electronic databases between February 2003 and February 2023: CNKI, Wanfang, VIP, China Biomedical Database, PubMed, Web of Science, Cochrane Library, Embase, and CINAHL.

**Figure 1:**
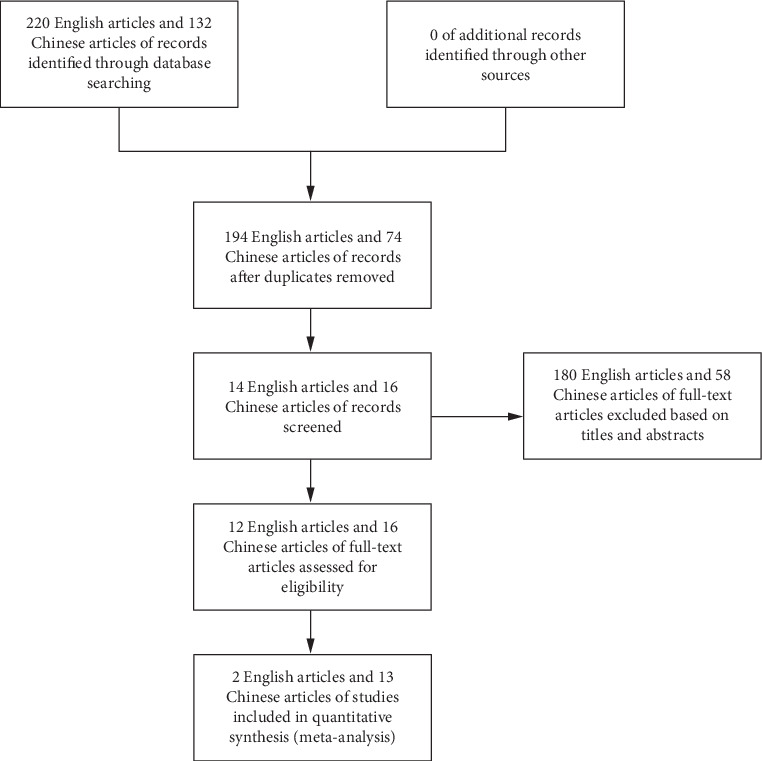
Flowchart of the selection process.

Using a combination of medical subject heading terms and free text, the search strategy for PubMed was as follows: (“aged” OR “old people” OR “elderly”) AND (“hip fracture” OR “femoral neck fractures” OR “femoral fractures” OR “osteoporotic fractures [MeSH Terms]” OR “femoral neck fractures [MeSH Terms]” OR “femoral fractures [MeSH Terms]” OR “hip fracture/rehabilitation [MeSH Terms]” OR “hip fractures/surgery∗ [MeSH Terms]”) AND (“frail∗” OR “frail syndrome/Syndrome∗” OR “Frailty”). To avoid the omission of the literature, references of the study were included in a snowball fashion.  Figure 1 depicts the flowchart of the screening and selection process.

### 2.2. Inclusion and Exclusion Criteria

The inclusion criteria were as follows: (1) the study population consisted of hip fracture patients, (2) the incidence of frailty and its influencing factors used as the outcome indicators, (3) case–control or cross-sectional or cohort studies, (4) assay method or survey tools explicitly mentioned in the literature, and (5) articles in English or Chinese. The exclusion criteria were as follows: (1) non-subject–related literature; (2) non-Chinese or English literature; (3) literature with incomplete information, data unable to convert, or unfull text; and (4) duplicate literature.

### 2.3. Literature Screening and Data Extraction

Two authors independently screened all citations and abstracts identified by the search strategy to select potentially eligible studies. In instances of disagreement, they consulted the original data for discussion or a third author to eliminate selection bias. Data were independently extracted from the included literature by two authors using a predesigned data extraction Excel file. The extracted information includes the first author of the included literature, study time, study location, study type, sample size, basic characteristics of the research object (source, age, etc.), frailty assessment tool, incidence of frailty, and evaluation indicators of influencing factors.

### 2.4. Quality Assessment

All eligible studies were subjected to a quality assessment by two authors independently. The Newcastle–Ottawa Scale (NOS) was used to evaluate the quality of the included case–control and cohort studies [18]. The NOS is a 9-point scale; studies were classified as low quality (0~4) and high quality (5~9). The cross-sectional studies were assessed by the observational research evaluation tool developed by the Agency for Healthcare Research and Quality (AHRQ); the tool contains 11 items with a total score of 11; studies were divided into three grades as low quality (0~3), medium quality (4~7), and high quality (8~11). The assessment results are shown in Tables 1 and 2.

### 2.5. Statistical Analysis

The prevalence of frailty in elderly hip fracture patients was calculated from available data from eligible studies. All statistical analyses were performed using Review Manager (RevMan) 5.3. In this meta-analysis, we calculated odds ratio (OR) and 95% CIs for morbidity. Heterogeneity among studies was tested using the *I*^2^ statistic and *χ*^2^ test. A significant heterogeneity between studies was inferred when *I*^2^ was equal to or greater than 50% (*p* < 0.05), prompting the use of a random-effects model for the meta-analysis. Otherwise, a fixed-effects model was utilized. For sensitivity analysis, a substitution effect model was applied to verify the robustness of the results via comparison and to identify the publication bias of the most frequently included influencing factors. If the funnel chart distribution of influencing factors is concentrated and symmetrical, it indicates that the publication bias of the study is well managed.

## 3. Results

### 3.1. Results of Literature Searching

A total of 352 reports were initially identified from the database and manual search. In the first phase of screening, 326 reports were excluded from the study for the following reasons: redundant publications, reviews, and unrelated topics. Subsequently, upon detailed examination of the remaining articles, 180 English articles and 58 Chinese articles of full text were excluded based on titles and abstracts. Ultimately, 15 papers were selected for inclusion in this research. The conditions in these studies and the clinical details of the patients are presented in Table 3.

### 3.2. Results of Included Literature

Our meta-analysis incorporated 15 articles, including 3475 elderly patients with hip fractures, out of which 1209 were identified as frail. The basic characteristics of the included studies are shown in Table 1. Five studies reported the prevalence of frailty, with two using different methods for frailty assessment. Seven studies discussed the factors contributing to frailty. In terms of frailty assessment tools, two studies used frailty index (FI); three studies used the frailty phenotype (FP); seven studies used FRAIL scale; two studies used the Edmonton Frailty Scale (EFS); one study used the modified frailty index (mFI), MFC, and Tilburg frailty scale. The methodological quality of all 15 studies was evaluated as medium grade, indicating a satisfactory level of rigor and precision in their research methodologies.

### 3.3. Results of Meta-Analysis

#### 3.3.1. Combination of Effect Value

**Figure 2:**
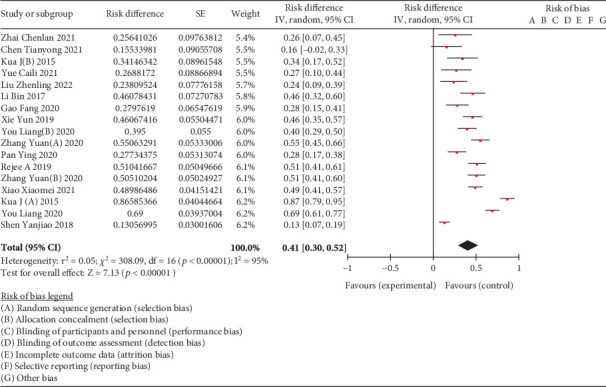
Combination of effect value of studies included.

A metaintegration was conducted on data extracted from 15 studies, focusing on the incidence of frailty among elderly patients with hip fractures, encompassing a total of 3475 cases. Heterogeneity test was executed using RevMan 5.3. The results indicated substantial heterogeneity across the studies (*I*^2^ = 95%, *p* < 0.00001). Despite this, the combined effect values were statistically significant, as shown in Figure 2.

#### 3.3.2. Subgroup Analysis

Twelve studies reported a frailty incidence of 44% (95% CI: 0.31~0.56) in 3048 hospitalized elderly patients with hip fractures and three studies reported a frailty incidence of 27% (95% CI: 0.19~0.35) in 427 elderly people with hip fractures. The results showed a significantly higher incidence of frailty among hospitalized patients compared to those in community settings (*χ*^2^ = 12.476, *p* < 0.001).

Meta-analysis of the random-effects model showed that 7 studies used FRAIL scale to measure the incidence of frailty in 1106 elderly patients with hip fractures which was 40% (95% CI: 0.32~0.49), 3 studies used FP scale to measure the incidence of frailty in elderly patients with hip fractures which was 39% (95% CI: 0.26~0.52), 2 studies used FI scale to measure the incidence of frailty in elderly patients with hip fractures which was 14% (95% CI: 0.14~0.96), and 2 studies used EFS to measure the incidence of frailty in old patients with hip fractures which was 44% (95% CI: 0.28~0.60); the studies used mFI, MFC, and Tilburg scales to measure the incidence of frailty in old patients with hip fractures which were 16% (95% CI: 0.02~0.33), 27% (95% CI: 0.10~0.44), and 87% (95% CI: 0.79~0.95).

### 3.4. Meta-Analysis Results of Factors Influencing Frailty in Elderly Patients With Hip Fracture

A meta-analysis was carried out on two studies to investigate the factors affecting frailty in elderly patients with hip fractures. The analysis showed no variations in the factors of age, duration of bed, and self-perceived health status; therefore, a fixed-effects model was employed. Variations were observed in the factors of comorbidity and nutritional status, so random-effects model was used. The results of meta-analysis showed that combined OR and 95% CI of age, comorbidity, duration of bed rest, nutritional status, and self-perceived health status were statistically significant, as shown in Table 4. Meta-analysis results showed that the risk factors for frailty in elderly with hip fractures were advanced age, comorbidity, prolonged bed rest, poor nutritional status, and poor self-perceived status. Other factors of frailty in elderly patients with hip fractures such as gender, the level of serum albumin (ALB), 25-(OH) VD (25-hydroxy vitamin D), deep vein thrombosis (DVT), handgrip strength, depression, and the mobility before fractures have not yet been synthesized in a meta-analysis.

### 3.5. Sensitivity Analysis and Publication Bias

**Figure 3:**
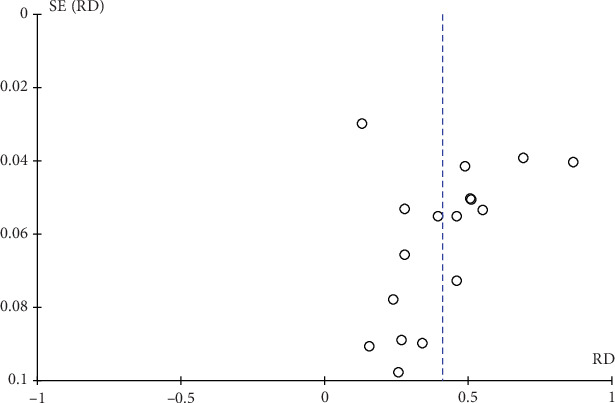
Funnel plot of the studies included.

The combined OR values of the included factors were estimated by fixed-effects model and random-effects model separately. The proximity of the results obtained from these two models substantiates the fundamental reliability of this study, as shown in Table 4. A funnel plot was used to assess the publication of potential publication bias within the included studies. And the publication bias of the included literatures was detected by observing the symmetry of funnel plot. As illustrated in Figure 3, no discernible publication bias was detected.

## 4. Discussion

This systematic review and meta-analysis assessed the prevalence and the impact of frailty in elderly hip fracture patients among 15 studies with relatively high methodological quality. Based on the 15 studies involving a total of 3475 patients, the estimated pooled prevalence of frailty in elderly hip fracture patients is 41%. However, it is crucial to interpret this finding with caution due to the considerable heterogeneity observed across the included studies. The findings of this study align with those of a previous meta-analysis, which reported an overall frailty prevalence of 41.4% among older patients in hospital settings [29]. This rate significantly exceeds the 12.8% frailty prevalence observed in the general elderly population [30]. The data underlines the necessity to prioritize the frailty condition in elderly hip fracture patients to mitigate potential negative health outcomes [31–34]. Several clinical guidelines of frailty management are recommended for early intervention on modifiable risk factors, including unhealthy diet and insufficient physical activity.

According to our results, the occurrence of frailty in hip fracture patients is strongly associated with factors such as older age, more comorbidity, prolonged bed rest, poor self-perceived health status, and poor nutritional status. Older age has been consistently identified as a significant factor associated with frailty, as evidenced by both exclusively physical-originated and comprehensive assessments. Three of these studies showed advanced age as an independent risk factor for frailty [10, 18, 19]. This study corroborates previous findings. Normally, aging of organs, exercise deficiency, and decrease of body resistance as well as body hormones decline, leading to physical limitations and psychological distress, thus resulting in an increased risk of frailty. It suggested that frailty risk may further impede an individual's social interactions and their capacity to adapt to environmental changes [35]. Therefore, medical staff should assess the frailty status of the elderly patients with hip fracture comprehensively and perform opportune therapeutic intervention to rectify any reversible debilitated conditions.

Comorbidity is a risk factor for frailty in elderly patients with hip fracture. Zhang et al. [28] demonstrated that there were many types of chronic diseases, and the incidence of frailty was higher in elderly patients with hip fragility fracture, which is consistent with the report by Morri et al. [36]. It has been substantiated that specific chronic diseases such as cancer, Type 2 diabetes, and depression, or their concurrent presence (i.e., multimorbidity), escalate the risk of frailty [26, 37, 38]. The presence of multiple comorbidities is capable of expediting the deterioration of physiological reserve functions across various organ systems. This leads to a persistent state of depletion, diminishing capacity for homeostasis, and disrupted internal equilibrium. Consequently, patients' tolerance decreases with susceptibility development, making them more prone to frailty. Patients with a large variety of comorbidities are under higher risk of disease-related complications after a hip fracture. As a consequence, detection rates of frailty in elderly hip patients with higher comorbidity are ascending. Considering that approximately 66% of older individuals have at least two chronic medical conditions [39, 40], it underlines effective strategy is needed to alleviate overall disease burden [41].

In patients with hip fractures, prolonged periods of bed rest are more likely to give rise to frailty. This is mainly due to the restriction of muscle activity in the lower limbs due to limb immobilization and pain. Basically, the preservation of muscle function relies on regular contraction and movement of the musculoskeletal system. When the muscle is in a braking or unloaded state, the muscle fibers would start to atrophy. Extended bed rest will probably result in reduced muscle protein synthesis, increased urinary nitrogen excretion, enhanced muscle decomposition, and weakness of lower limb muscle strength, thus inducing the occurrence of weakness [42]. Research has shown that in elderly patients, undermobility can lead to a 10% decrease in lower skeletal muscle mass within 10 days, while inpatient limb immobilization can result in more than a 10% drop downward trend within only 3 days [43]. Our data suggests that prolonged bed rest is an independent risk factor for frailty after surgery for hip fracture. Exercise has once again proven to be a very effective intervention in improving frailty in elderly people [44]. Therefore, even if postoperative weight-bearing activities are not recommended, functional exercise should be performed as early as possible to maintain muscle mass or reduce muscle atrophy and then prevent the occurrence of frailty.

Nutritional status influences frailty in elderly patients with hip fracture. Scholar Alfonso [45] claimed that malnutrition plays an important role in the pathogenesis of frailty and can contribute to its development through energy, protein, creatine, and other nutrients. Malnutrition is able to cause abnormal immune system function in elderly patients following postoperative hip fracture, resulting in the impairment of organ function as well as the decline of physiological function, and then induce frailty [46, 47]. At the same time, nutrition intervention is also a crucial strategy to prevent and slow down the progression of frailty [48]. In light of this, it is suggested that elderly patients with hip fractures and poor nutritional status should be improved to enhance their postoperative recovery. This would ensure an improvement in their nutritional status and consequently reduce the incidence of frailty. In this study, it indicates that self-perceived health status is a protective factor for frailty in elderly patients with hip fracture, and poor self-perceived health status is associated with a higher prevalence of frailty in hip fracture elderly patients, corroborating the report by Curcio et al. and Lo Buglio et al. [49, 50]. Additionally, when elderly patients have negative views of adverse experiences in life, they often have negative self-protection mechanisms, potentially leading to health complications that may precipitate frailty [24, 51]. It has been proved that self-perceived health condition exhibits convenient operation and good credit validity, providing a good indication of health as well as a reliable predictor of mortality [52, 53]. Consequently, healthcare professionals and particularly nurses should attach great importance to self-perceived health condition of the elderly patients with hip fracture. Hence, prioritizing intervention strategies to enhance individual health status is fundamental, guiding patients and their families into proper cooperation with treatment plans.

As there is paucity of result consistency, there are relatively few studies on influencing factors such as gender, hand grip strength, and family care on frailty in elderly hip fracture patients. Therefore, further research is warranted to elucidate the relationship between these variables and frailty in this patient population.

## 5. Limitations and Future Directions

Our study had some limitations. There is substantial heterogeneity among the included studies. However, heterogeneity is often unavoidable in meta-analyses of observational studies; thus, it does not necessarily invalidate our findings. Most of the included studies are cross-sectional, with few cohort studies and case–control studies, because of the limited availability of the design type. Hence, more clinical studies should be conducted and evaluated in the future. There is no unified screening tool for frailty, which will have an impact on the incidence and influencing factors. Most of the studies in this review were conducted in China, only two studies were from the United Kingdom and Singapore, and more studies from diverse countries are needed to explore this topic. Another limitation is the difference in methods used to describe and classify fracture patterns. Different criteria were used for classification. Further analysis of fracture characteristics within the included studies may have provided some additional benefit. In summary, our findings underscore the importance of routine screening programs to evaluate the frailty of elderly patients with hip fractures, thereby facilitating the prompt implementation of appropriate interventions.

## 6. Conclusion

This systematic review identified multiple factors influencing frailty in elderly patients with hip fractures, including advanced age, heightened comorbidity, prolonged bed rest, poor self-perceived health status, and poor nutritional condition. These findings underline the imperative work for healthcare professionals to prioritize the consideration of influencing factors, conduct regular comprehensive evaluations, and implement appropriate interventions tailored to these factors to prevent and decelerate the progression of frailty.

## Figures and Tables

**Table 1:** Risk of bias evaluation levels for cross-sectional studies.

**Study**	**①**	**②**	**③**	**④**	**⑤**	**⑥**	**⑦**	**⑧**	**⑨**	**⑩**	**⑪**	**Total score**
Xie et al. [19]	Yes	Yes	Yes	No	No	No	No	Yes	No	Yes	Yes	7
Zhai, Liu, and Shi [11]	Yes	Yes	Yes	No	No	No	No	Yes	No	No	No	5
Liu et al. [20]	Yes	Yes	Yes	No	No	No	No	Yes	No	Yes	No	6
Yue [12]	Yes	Yes	Yes	No	No	No	No	Yes	No	No	Yes	6
Pan and Li [13]	Yes	Yes	Yes	No	Yes	No	No	Yes	No	No	Yes	7
Gao [21]	Yes	Yes	Yes	No	No	Yes	Yes	Yes	No	No	No	6
Shen [22]	Yes	Yes	Yes	No	No	No	No	Yes	No	No	No	5
You [23]	Yes	Yes	Yes	No	No	No	No	Yes	No	No	Yes	6
You [23]	Yes	Yes	Yes	No	No	No	No	Yes	No	No	Yes	6
Zhang et al. [6]	Yes	Yes	Yes	No	No	No	No	Yes	No	Yes	No	6
Li et al. [24]	Yes	Yes	Yes	No	Yes	Yes	No	Yes	No	Yes	No	7
Xia [25]	Yes	Yes	Yes	No	No	No	No	Yes	No	No	No	5
Chen, Xuan, and Wu [26]	Yes	Yes	Yes	No	No	No	No	Yes	No	Yes	No	6

*Note:* ①: whether the source of information was specified (survey, literature review); ②: whether inclusion and exclusion criteria for exposed and nonexposed groups (cases and controls) are listed or reference to previous publications; ③: whether the time stage was given to identify patients; ④: whether the study population is continuous if it is not a population source; ⑤: whether the evaluator's subjective factors obscure other aspects of the subject's condition; ⑥: describes any assessments conducted for quality assurance (e.g., testing/retesting of key outcome indicators); ⑦: explained the rationale for excluding any patient from the analysis; ⑧: describes how to evaluate and/or control measures for confounding factors; ⑨: if possible, explains how lost data is handled in the analysis; ⑩: summarized the response rate of patients and the completeness of data collection; ⑪: if follow-up is available, identify the percentage of patients with incomplete data expected or the outcome of the follow-up.

**Table 2:** Results of risk of bias evaluation for case–control and cohort studies (scores).

**Study**	**Selection of study subjects**	**Component comparability**	**Exposure factors/outcome measures**	**Total score**
Chen, Xuan, and Wu [26]	4	1	1	6
Rajeev and Anto [27]	3	1	3	7
Kua et al. [10]	4	1	2	7

**Table 3:** Basic characteristics of the included studies.

**Study**	**Study type**	**Location**	**Sample source**	**Sample size (M/F)**	**Age**	**Measure tool**	**Amount of frailty**	**Influencing factors**
Xie et al. [19]	Cross-sectional studies	Sichuan, China	Hospital	178 (64/114)	≥ 65	①	82	
Rajeev and Anto [27]	Case–control study	Zhejiang, China	Hospital	103 (40/63)	≥ 65	②	16	
Zhai, Liu, and Shi [11]	Cross-sectional studies	Henan, China	Community	78 (35/43)	≥ 60	①	20	1, 3, 4, 6, 7
Liu et al. [20]	Cross-sectional studies	Jiangxi, China	Hospital	126 (68/48)	≥ 60	①	30	1, 3, 8, 9, 10
Yue [12]	Cross-sectional studies	Henan, China	Community	93 (60/33)	≥ 60	③	25	7, 10, 15, 16
Pan and Li [13]	Cross-sectional studies	Jiangsu, China	Community	256 (90/166)	≥ 60	④	71	2, 3, 7, 17
Gao [21]	Cross-sectional studies	Sichuan, China	Hospital	168 (65/103)	≥ 60	①	47	1, 3, 9, 11
Shen [22]	Cross-sectional studies	Sichuan, China	Hospital	965 (328/637)	≥ 60	⑤	126	
You [23]	Cross-sectional studies	Jiangxi, China	Hospital	200 (96/104)	≥ 60	⑤	138	3
You [23]	Cross-sectional studies	Jiangxi, China	Hospital	200 (96/104)	≥ 60	④	79	
Zhang et al. [6]	Cross-sectional studies	Xinjiang, China	Hospital	158 (60/198)	≥ 60	①	87	3, 5, 12, 13
Li et al. [24]	Cross-sectional studies	Xinjiang, China	Hospital	196 (68/128)	≥ 60	④	99	1, 3, 4, 5, 14
Xia [25]	Cross-sectional studies	Beijing, China	Hospital	102 (35/67)	≥ 65	①	47	
Chen, Xuan, and Wu [26]	Cross-sectional studies	China	Hospital	296 (64/114)	≥ 60	①	145	
Zhang et al. [28]	Case-control study	United Kingdom	Hospital	192	≥ 60	⑥	98	3
Kua et al. [10]	Control study	Singapore	Hospital	82 (27/55)	≥ 60	⑦, ⑧	71/28	1, 2

*Note:* Measure tool: ①: FRAIL scale; ②: mFI scale; ③: Tilburg scale; ④: FP scale; ⑤: FI scale; ⑥: Edmonton Frailty Scale (EFS); ⑦: Modified Fried Criteria (MFC); ⑧: Reported Edmonton Frail Scale (REFS). Influencing factors: 1—age; 2—gender; 3—comorbidity; 4—nutritional status; 5—self-perceived health status; 6—nearly 1 year fall history; 7—postoperative bed rest time; 8—preoperative anemia; 9—postoperative functional exercise start time; 10—albumin serum level; 11—mobility before fractures; 12—depression; 13—handgrip strength; 14—quality of sleep; 15—25-OH VD; 16—family care degree; 17—DVT.

**Table 4:** Heterogeneity tests and meta-analysis results of factors influencing frailty in elderly patients with hip fractures.

**Influencing factors**	**Amount of included studies**	**Heterogeneity test**		**Effect model**	**Combined OR value (95% CI)**		**Effect size test**	
		**p**	**I** ^2^		**OR**	**95% CI**	**Z**	**p**
Ages	4	*p* = 0.63	0%	Fixed	2.73	[2.12, 3.53]	7.72	*p* < 0.00001
Comorbidity	5	*p* < 0.00001	90%	Random	4.20	[2.31, 7.62]	4.71	*p* < 0.00001
Duration of bed best	3	*p* = 0.71	0%	Fixed	2.22	[1.54, 3.18]	4.32	*p* < 0.0001
Nutritional status	2	*p* = 0.03	78%	Random	1.62	[1.21, 2.17]	3.23	*p* = 0.001
Self-perceived health status	2	*p* = 0.22	32%	Fixed	3.53	[2.09, 5.94]	4.73	*p* < 0.00001

## Data Availability

The data sets used and analyzed during the current study are available from the corresponding authors on reasonable request.
